# The Recombinant Protein Based on *Trypanosoma cruzi* P21 Interacts With CXCR4 Receptor and Abrogates the Invasive Phenotype of Human Breast Cancer Cells

**DOI:** 10.3389/fcell.2020.569729

**Published:** 2020-10-19

**Authors:** Bruna Cristina Borges, Isadora Akemi Uehara, Marlus Alves dos Santos, Flávia Alves Martins, Fernanda Carvalho de Souza, Álvaro Ferreira Junior, Felipe Andrés Cordero da Luz, Mylla Spirandelli da Costa, Ana Flávia Oliveira Notário, Daiana Silva Lopes, Samuel Cota Teixeira, Thaise Lara Teixeira, Patrícia de Castilhos, Claudio Vieira da Silva, Marcelo José Barbosa Silva

**Affiliations:** ^1^Laboratório de Tripanosomatídeos, Departamento de Imunologia, Instituto de Ciências Biomédicas, Universidade Federal de Uberlândia, Uberlândia, Brazil; ^2^Laboratório de Biomarcadores Tumorais e Osteoimunologia, Departamento de Imunologia, Instituto de Ciências Biomédicas, Universidade Federal de Uberlândia, Uberlândia, Brazil; ^3^Departamento de MedicinaVeterinária, Escola de Veterinária e Zootecnia, Universidade Federal de Goiás, Goiânia, Brazil; ^4^Laboratório de Nanobiotecnologia, Instituto de Genético e Bioquímica, Universidade Federal de Uberlândia, Uberlândia, Brazil; ^5^Instituto Multidisciplinar em Saúde, Universidade Federal da Bahia, Vitória da Conquista, Brazil; ^6^Laboratório de Biologia Molecular de Trypanosoma Cruzi, Departamento de Parasitologia, Universidade Federal de São Paulo, São Paulo, Brazil

**Keywords:** *Trypanosoma cruzi*, recombinant protein P21, CXCR4, triple-negative breast cancer cells, invasion cell

## Abstract

*Trypanosoma cruzi* P21 is a protein secreted by the parasite that plays biological roles directly involved in the progression of Chagas disease. The recombinant protein (rP21) demonstrates biological properties, such as binding to CXCR4 receptors in macrophages, chemotactic activity of immune cells, and inhibiting angiogenesis. This study aimed to verify the effects of rP21 interaction with CXCR4 from non-tumoral cells (MCF-10A) and triple-negative breast cancer cells (MDA-MB-231). Our data showed that the MDA-MB-231 cells expressed higher levels of CXCR4 than did the non-tumor cell lines. Besides, cytotoxicity assays using different concentrations of rP21 showed that the recombinant protein was non-toxic and was able to bind to the cell membranes of both cell lineages. In addition, rP21 reduced the migration and invasion of MDA-MB-231 cells by the downregulation of *MMP-9* gene expression. In addition, treatment with rP21 blocked the cell cycle, arresting it in the G1 phase, mainly in MDA-MB-231 cells. Finally, rP21 prevents the chemotaxis and proliferation induced by CXCL12. Our data showed that rP21 binds to the CXCR4 receptors in both cells, downregulates CXCR4 gene expression, and decreases the receptors in the cytoplasm of MDA-MB-231 cells, suggesting CXCR4 internalization. This internalization may explain the desensitization of the receptors in these cells. Thus, rP21 prevents migration, invasion, and progression in MDA-MB-231 cells.

## Introduction

Triple-negative breast cancer (TNBC) is characterized by a tumor subtype void of hormone receptors, such as the estrogen receptors (ER), progesterone receptors (PR), and human epidermal growth factor receptor 2 (HER2) (Zhou et al., 2018); thus, this tumor is associated with poor prognosis (Den Brok et al., 2017). Poor prognosis involves a distinct metastatic pattern involving regional lymph nodes, bone marrow, the lungs, and liver (Müller et al., 2001) and ineffective treatments owing to the lack of therapeutic targets (Venkitaraman, 2010; Voduc et al., 2010).

Most deaths caused by breast cancer are not due to the primary tumor itself but are a result of metastasis to other organs in the body (Weigelt et al., 2005). Chemokines and receptors regulate tumor cell migration and metastasis. The CXCR4 receptor is a seven-transmembrane domain G-protein-coupled receptor (GPCR) superfamily member (Cojoc et al., 2013). CXCL12 is a chemokine that binds to this receptor. The CXCR4/CXCL12 axis can promote tumor metastasis by mediating cell invasion and proliferation and also enhancing tumor-associated neoangiogenesis (Cojoc et al., 2013). It is involved in orientating cancer cell migration to metastasis sites, increased survival of cancer cells in suboptimal conditions, and the establishment of a tumor-promoting cytokine/chemokine network (Balkwill, 2004). This receptor is expressed constitutively in a wide variety of normal tissues, including lymphatic tissues, thymus, brain, spleen, stomach, and small intestine, but it is also expressed in several types of tumor cells (Balkwill, 2004). Tumor cells increase the CXCR4 levels and CXCL12 production, transmitting autocrine and paracrine signals, leading to enhanced tumor growth and metastasis (Liekens et al., 2010).

Microorganisms and viruses can contribute to cancer initiation and progression (Whisner and Aktipis, 2019), such as *Helicobacter pylori* in stomach cancer (Martel et al., 2008), *Herpes papillomavirus* in cervical cancer (Schiffman et al., 2007), and hepatitis C or B in liver cancer (Chan et al., 2016; Axley et al., 2018). Several studies involving parasites demonstrate that bioactive molecules and parasites promote antitumor effects, such as *Strongyloides stercoralis*, *Toxoplasma gondii*, *Plasmodium*, and *Trypanosoma cruzi* (Plumelle et al., 1997; Kim et al., 2007; Chen et al., 2011; Lukasiewicz and Fol, 2018). It has been demonstrated that parasite anticancer activity is mediated by antitumor immunity induction and immunomodulation (Ubillos et al., 2016; Mohamadi et al., 2019; Riaz, 2019), gene regulation (Lu et al., 2019), and anticancer effects by parasite molecule production (Atayde et al., 2008; Valck et al., 2010; Darani and Yousefi, 2012; Ramírez et al., 2012).

Different strains of *T. cruzi* were used for carcinoma treatment and showed that high parasitemia is related to a decreased tumor development in animal models (Krementsov, 2009), and parasite extracts had the same effect (Krementsov, 2009). Thus, the immune response elicited by *T. cruzi* could be effective toward tumor cells due to the molecular mimicry of antigens (Zhigunova et al., 2013; Ubillos et al., 2016). Besides that, it is known that *T. cruzi* has a component with pro-apoptotic activity in tumor cells (Mucci et al., 2006) and antitumor membrane proteins, such as GP82 and calreticulin protein (Atayde et al., 2008; Valck et al., 2010; Ramírez et al., 2012).

P21 is a *T. cruzi* protein involved in parasite–host cell invasion and parasite perpetuation during infection (Silva et al., 2009). Some results have shown that the recombinant form of this protein (rP21) acts as a phagocytosis inducer by binding to the CXCR4 chemokine receptor and activating actin polymerization in macrophages (Rodrigues et al., 2012). This recombinant protein can also increase sFlt-1 production by macrophages. This soluble molecule inhibits endothelial cell proliferation, ensuring an anti-angiogenic action (Teixeira et al., 2015; Teixeira et al., 2017); besides that, rP21 can promote the chemotaxis of immune cells (Teixeira et al., 2015). In this way, it is interesting to consider novel studies exploring rP21 in the tumoral microenvironment. This study aimed to evaluate the effects of the rP21 protein on breast cancer cells *in vitro*.

## Materials and Methods

### Cell Culture

Non-tumorigenic human breast cells (MCF-10A) and human triple-negative breast tumoral cells (MDA-MB-231) were purchased from Banco de Células do Rio de Janeiro. MCF-10A cells were cultivated in Dulbecco’s modified Eagle’s/Ham’s Nutrient Mixture F12 (DMEM/F12; Life Technologies, Carlsbad, CA, United States) supplemented with epidermal factor growth (20 ng/ml), insulin from bovine pancreas (10 μg/ml), hydrocortisone (0.5 μg/ml), and 5% fetal bovine serum (FBS). MDA-MB-231 cells were cultivated in DMEM medium (Sigma-Aldrich, MO, United States) supplemented with 10% FBS and 2 mM sodium bicarbonate. Both cells were maintained with 100 U/ml penicillin and 100 μg/ml streptomycin and incubated at 37°C in a humidified atmosphere containing 5% CO_2_.

### Production and Purification of rP21

The rP21 (GenBank: EU004210.1) protein was purified as previously described (Silva et al., 2009; Santos et al., 2014). After purification, the protein was incubated with a polymyxin B column. The quality of purification was demonstrated by sodium dodecyl sulfate–polyacrylamide gel electrophoresis (SDS-PAGE) and Western blotting (Supplementary Figure 1).

### Flow Cytometry

To determine CXCR4 levels, the cells were fixed with 4% paraformaldehyde for at least 1 h and permeabilized with saponin PGN, and stained cells were labeled with PE anti-human CD184 antibodies (BioLegend, CA, United States) for 1 h at 4°C. Data from 12,000 cells were collected by a CytoFLEX (Beckman Coulter). Results were obtained using Kaluza software (Beckman Coulter). The mean fluorescence intensities (MFI) were calculated using the median of samples on a logarithmic scale.

The binding of rP21 to cells was determined. The cells (1 × 10^5^) were treated with rP21 (100 μg/ml) for 1 h. Next, they were washed two times in phosphate-buffered saline (PBS). The protein was stained using primary IgY anti-rP21 antibodies diluted in 1:10 in 2.5% bovine serum albumin (BSA) for 1 h. The cells were washed twice and secondary anti-IgY fluorescein isothiocyanate (FITC) antibodies were diluted 1:1,000 in 2.5% BSA for 1 h. After this, the samples were collected in the BD FACSVerse (Becton Dickinson, United States). The results were obtained using FlowJo software (FlowJo, LLC).

To verify whether rP21 binding to cells interferes with the CXCR4 receptors, cells (1 × 10^5^) were treated for 1 and 72 h (100 μg/ml rP21). For labeling of the CXCR4 receptor in the membrane, after treatment, the cells were washed and labeled with PE anti-human CD184 antibodies diluted in 2.5% BSA solution (3 μl of antibody in 100 μl of solution). To label the CXCR4 in the cytoplasm, the cells were fixed with 4% paraformaldehyde for 1 h after the PE anti-human CD184 antibodies were diluted in saponin PGN (saline buffer with gelatin) for 1 h at 4°C. Data from 12,000 cells were collected by a CytoFLEX (Beckman Coulter). The results were obtained using Kaluza software (Beckman Coulter).

### Confocal Microscopy

To detect CXCR4, 1 × 10^5^ cells were seeded in 24-well coverslips. Next, the cells were fixed with 4% formaldehyde for 1 h and washed three times with PBS. Then, they were permeabilized and blocked with saponin PGN and labeled with PE anti-human CD184 antibodies (BioLegend, CA, United States) for 1 h at 4°C. The images qualitatively show the CXCR4 levels.

To analyze the co-localization between rP21 and CXCR4, 1 × 10^5^ cells were seeded in 24-well coverslips and treated at 1, 6, and 24 h with 100 μg/ml rP21. The cells were stained with primary antibody IgY anti-rP21 overnight. Next, the secondary antibody anti-IgY FITC, PE anti-human CD184 antibodies, and DAPI were added. Images were obtained in a Zeiss LSM 510 META microscope at × 63 magnification.

### Quantitative Reverse Transcription PCR

MDA-MB-231 and MCF-10A (1 × 10^4^ cells/well) were seeded in 24-well microplates that had previously been coated with a thin Matrigel. The cells were incubated with rP21 (100 μg/ml), CXCL12 (20 ng/ml), or culture medium (control group) for 72 h. Total RNA was extracted using a Maxwell^®^ RSC simplyRNA Cells Kit (Promega, Madison, WI, United States) and GoScript RT Mix (Promega) was used for reverse transcription according to the manufacturer’s instructions. Quantitative RT-PCR was performed using a GoTaq^®^ Master Mix qPCR (Promega) and StepOnePlus^TM^ (Applied Biosystems, Carlsbad, CA, United States) was used to analyze the received data. The data were normalized using HPRT-1 as a housekeeping gene and CXCR4 and MMP9 genes were analyzed. The primers used were: CXCR4, 5′-GGGATCAGTATATACACTTCAGATA-3′ (forward) and 5′-GCTGTGACCTGCTGTTATT-3′ (reverse); MMP-9, 5′-AAGGACCGGTTCATTTGG-3′ (forward) and 5′-CCTCGTATACCGCATCAATC-3′ (reverse); and HPRT-1, 5′-GGCGTCGTGATTAGTGATG-3′ (forward) and 5′-AACACCCTTTCCAAATCCTC-3′ (reverse). The analysis was done by the comparative threshold cycle (*C*_T_) method to calculate fold changes in expression in treated groups compared to the control group. The fold changes in gene expression for the treated groups were then calculated as 2^–ΔΔCT^.

### Viability Assay

The effect of rP21 on TNBC and normal cells was determined using the 3-[4,5-dimethylthiazol-2-yl]-2,5-diphenyl tetrazolium bromide (MTT) assay. The cells (1 × 10^4^) were seeded in 96-well plates. After adhesion, the cells were treated with different concentrations of rP21 (6.25, 12.5, 25, 50, and 100 μg/ml) for 72 h. Afterward, the cells were incubated with 10 μl MTT (5 mg/ml) per well for 4 h. The dye crystals were dissolved in dimethylformamide (50%) containing 10% SDS overnight. Absorbance was measured at 570 nm on the multiwell scanning spectrophotometer GloMax Explorer (Promega, United States). Cell viability was expressed in percentage, which was calculated as follows: (%) = [(absorbance of the control group – absorbance of the test group)/absorbance of the control group] × 100%. Concentrations that present less than 50% of viable cells would be considered toxic.

### Migration/Invasion Assay

A wound healing assay was made by migration analysis. The cells were cultured as confluent monolayers for 24 h and wounded by removing a strip of cells across the well with a standard 10-μl pipette tip. Wounded monolayers were washed twice to remove non-adherent cells. Then, they were treated for 72 h with only the culture medium, 20 ng/ml CXCL12, and 100 μg/ml rP21 and pretreated with 100 μg/ml rP21 for 1 h and then treated with 20 ng/ml CXCL12. Wound healing was quantified using ImageJ software as the mean percentage of the remaining cell-free area compared to the area of the initial wound.

Invasion assay was made using transwells with 8-μm pores (Costar, Corning, United States) with Matrigel (Corning^®^ Matrigel^®^ Growth Factor Reduced). The upper chamber contained cells in the culture medium (1 × 10^5^/ml) and the lower chamber contained the culture medium (negative control), 20 ng/ml CXCL12 (chemoattractant), and 100 μg/ml rP21. In one group, the cells were pretreated with 100 μg/ml rP21 for 1 h and then added in the upper chamber. The lower chamber contained 20 ng/ml CXCL12 (chemoattractant). The cells were incubated for 72 h at 37°C in 5% CO_2_. Non-migrated cells were scraped from the upper surface of the membrane with a cotton swab and the migrated cells remaining on the bottom surface were counted after staining with crystal violet. Cell counting was done using a Leica DM 500 microscope at × 10 magnification. The images were used to count the number of cells using ImageJ software.

### Cell Cycle Assay

For cell cycle analysis, 7 × 10^4^ cells were seeded in 24-well plates. After 24 h, the cells were treated with culture medium, 20 ng/ml CXCL12, and 100 μg/ml rP21 and pretreated with 100 μg/ml rP21 for 1 h and then 20 ng/ml CXCL12 for 72 h. Then, they were collected, washed once with PBS, and fixed with 70% ethanol at 20°C for 24 h. Fixed cells were washed three times and incubated for 45 min with propidium iodide (PI) solution (10 μg/ml) containing RNase A (0.1 μg/ml). The cells were analyzed for determining DNA contents by flow cytometry. Cell debris was excluded based on forward vs. side scatter. Data from 12,000 events were collected in the final gated histograms. The cell histogram was divided into three regions according to the cell cycle phase: G1, S, and G2/M. Cells before the row represent unstained events. The inhibition percentage of the cell cycle was calculated as follows: (% inhibition) = (% G1 treated with rP21 –% G1 treated with CXCL12).

### Statistical Analysis

All data are presented as the mean ± standard error of the mean of experiments performed at least three times in triplicate. All data were first checked for normal distribution. Significant differences were determined by one-way ANOVA, Tukey’s multiple comparisons test, and Student’s *t*-test (two-sided) for parametric data or the Mann–Whitney test for non-parametric data according to the experimental design. *P* < 0.05 was considered significant. All the statistical analyses were performed using GraphPad Prism software version 8.0.

## Results

### CXCR4 Has a Distinct Amount in Non-tumoral and MDA-MB-231 Cells and rP21 Was Not Cytotoxic and Binds in Both Cells

**FIGURE 1:**
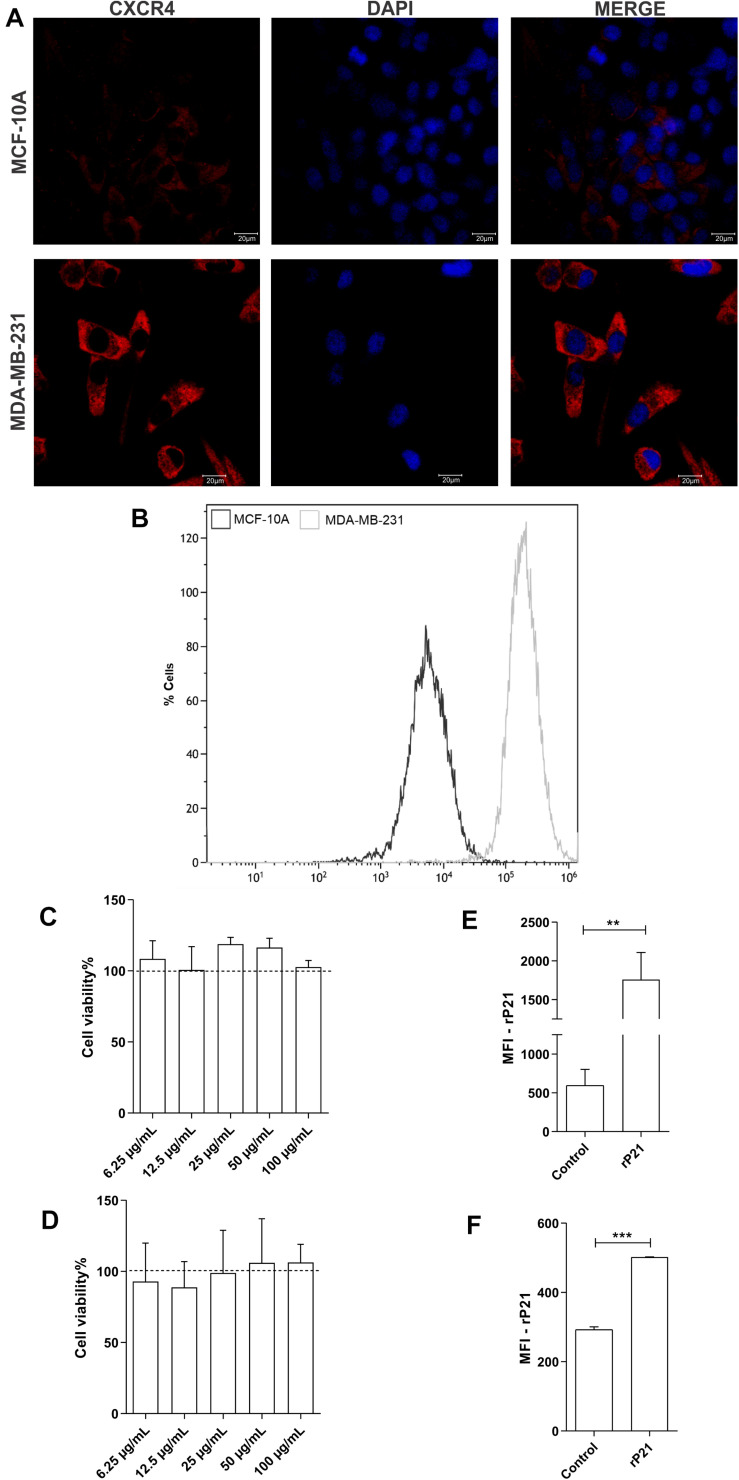
Differential expression of CXCR4 in membrane cells and total receptors in MDA-MB-231 and MCF-10A. Recombinant protein (rP21) is not cytotoxic and binds in cells. Evaluation of CXCR4 levels by confocal microscopy **(A)** and flow cytometry **(B)**. MCF-10A **(C)** and MDA-MB-231 **(D)** were treated with rP21 at different concentrations (100, 50, 25, 12.5 and 6.25 μg/mL) and did not exhibit alterations in cell viability. These data are from one experiment representative of three independent experiments. The cells were incubated for 1 hour with rP21 (100 μg/mL). rP21 labeling by flow cytometry showed protein binding in the MCF-10 A **(E)** and MDA-MB-231 **(F)** cells. These results are representative of at least three independent experiments. Data show the mean ± SEM. Significant differences were determined using student *t*-tests and one-way ANOVA. Differences were considered significant when *p* < 0.05. **p = 0.0025, ***p = 0.0005, and ****p < 0.0001.

First, we evaluated total CXCR4 levels in the plasma membrane and cytoplasm by confocal microscopy and CXCR4 on the cell surface by flow cytometry. Our data demonstrated higher labeling of the CXCR4 receptors in MDA-MB-231 cells than in MCF-10A cells, and MDA-MB-231 showed higher MFI values than did MCF-10A (Figures 1A,B).

Then, MCF-10A and MDA-MB-231 cells were treated with 100, 50, 25, 12.5, or 6.25 μg/ml rP21 for 72 h followed by the MTT assay to determine the effect of rP21 on cell viability. MCF-10A (Figure 1C) and MDA-MB-231 (Figure 1D) viability did not change with treatment.

As long as the cell lines expressed CXCR4, we addressed the ability of the rP21 protein to bind to the plasma membranes of these cell lineages. The data showed that rP21 adhered to MCF-10A (Figure 1E) and MDA-MB-231 membranes (Figure 1F) after treatment for 1 h.

### rP21 Modulates the Migration and Invasion of MDA-MB-231 by MMP-9 Downregulation

**FIGURE 2:**
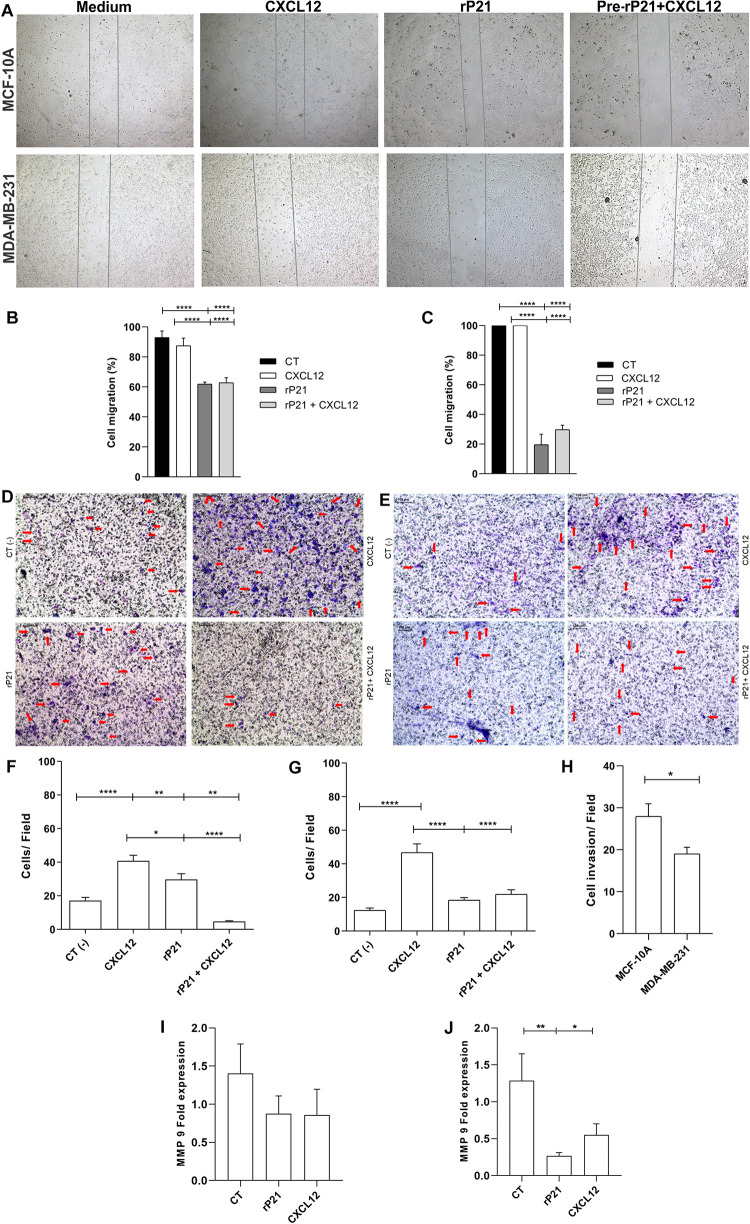
The rP21 protein decreased the migration and invasion of triple-negative breast cancer (TNBC) cells by downregulating MMP-9 gene expression. **(A)** Representative images show the differences in cell migration at 72 h in the wound healing assay. Percentages of MCF-10A **(B)** and MDA-MB-231 **(C)** cell closure of the wound healing assay area after treatments. Transwell cell invasion of MCF-10A **(D,F)** and MDA-MB-231 **(E,G)**. The percentage of cells invaded was determined using CXCL12 as 100%. Negative control: serum-free medium; positive control: medium 17 containing CXCL12 **(H)**. Bars, 100 μm represents × 10 objective. Red arrows indicate invaded cells. Gene expressions of MMP-9 in MCF-10A **(I)** and MDA-MB-231 **(J)**. These results are representative of at least three independent experiments. Data show the mean ± SEM. Significant differences were determined using one-way ANOVA and Tukey’s multiple comparisons test. Differences were considered significant when *p* < 0.05. *p < 0.05, **p < 0.01, and ****p < 0.0001.

Our next goal was to analyze the potential of rP21 to induce the migration and invasion of these cell lines. For this, the wound healing and Transwell assays were performed (Figure 2A). In the wound healing assay, treatment with rP21 for 72 h reduced the invasion of MCF-10A cells when compared to treatment with CXCL12 (Figure 2B). However, the reduction in invasion was much more expressed in MDA-MB-231 in the presence of rP21 (Figure 2C). In addition, when we pretreated the cells with rP21 and then added CXCL12, the inhibition of migration caused by rP21 was not altered in both cells (Figures 2B,C).

In the Transwell assay, treatment with rP21 as well as pre-incubation with rP21, and the subsequent addition of CXCL12, reduced cell invasion in MCF-10A (Figures 2D,F), but was further reduced in MDA-MB-231 (Figures 2E,G). Similar to the migration test, in this one, rP21 also prevented the chemotactic action of CXCL12. These data were more evident when comparing treatment with rP21 and the negative control. The invasion of MCF-10A cells was high (Figure 2F), while the invasion of MDA-MB-231 was less than that of the negative control (Figure 2G). Thus, in both cells, the invasion is smaller than that the CXCL12 group, but the invasion percentage of MDA-MB-231 is smaller than that in MCF-10A (Figure 2H).

We proved that rP21 can decrease the migration and invasion, mainly in MDA-MB-231. To assess this effect, matrix metalloproteinase 9 (MMP-9) expression was analyzed. In MCF-10A cells, MMP-9 gene expression remained similar between the control and treatment groups (Figure 2I), but in MDA-MB-231, rP21 treatment significantly decreased MMP-9 expression and CXCL12 slightly decreased MMP-9 expression (Figure 2J).

### rP21 Arrested Cell Cycle of MDA-MB-231 Cell Progression

**FIGURE 3:**
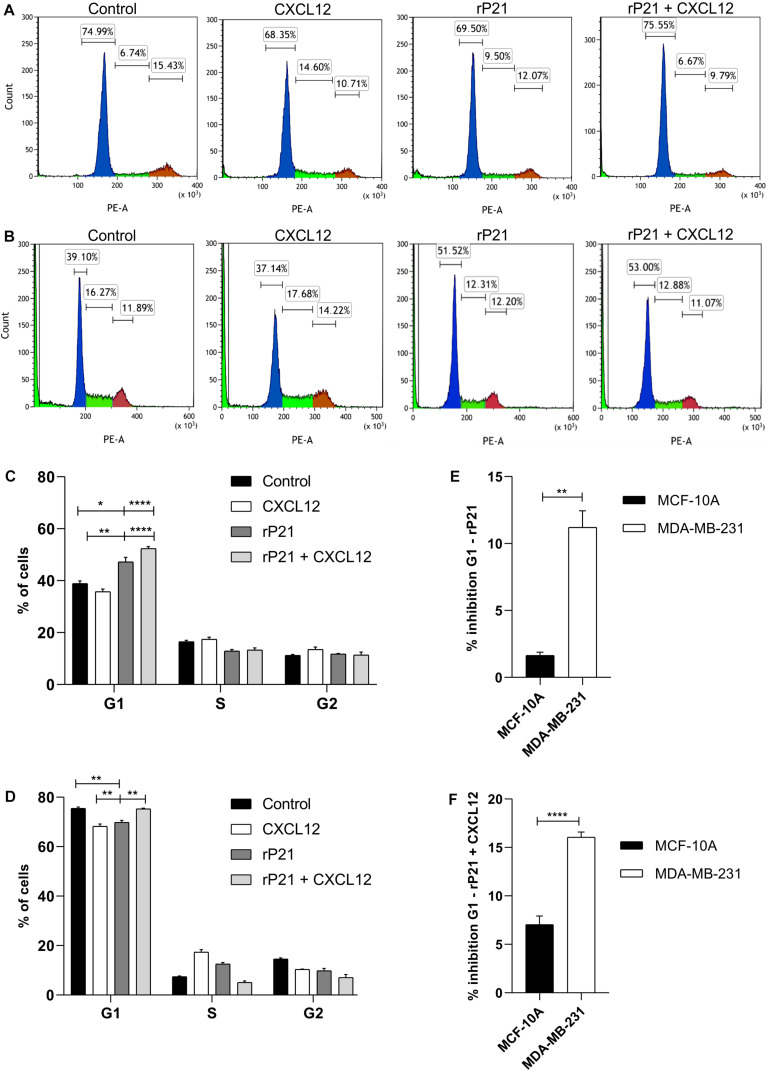
The rP21 protein caused G1 to be arrested in the MDA-MB-231 cell cycle. The DNA content of MCF-10A and MDA-MB-231 cells treated with different treatments at 72 h was analyzed by propidium iodide (PI) staining. Treatments: culture medium (control), CXCL12, rP21, rP21 for 1 h, and followed by CXCL12. Percentages of MCF-10A **(A,C)** and MDA-MB-231 **(B,D**) cells in each cell phase at different treatments. Inhibition percentages of the cell cycle in the rP21 treatment **(E)** and rP21 pretreatment followed by CXCL12 **(F)**. These results are representative of at least three independent experiments. Values are the mean ± SEM. Significant differences were determined using one-way ANOVA and Tukey’s multiple comparisons test. Differences were considered significant when *p* < 0.05. *p < 0.05, **p < 0.01, and ****p < 0.0001.

We further characterized the effects of rP21 on cell cycle. The percentages of MCF-10A and MDA-MB-231 cells in each cycle phase in different treatments are shown in Figures 3A,B, respectively. rP21 treatment in MCF-10A decreased cells in the G1 phase (69.50%), while rP21 pretreatment followed by CXCL12 kept MCF-10A cells equal to the control (75.55%; Figures 3A,C). Thus, rP21 treatment or pretreatment does not interfere in the cell cycle when compared to the control. When compared with CXCL12, the rP21 protein increases the cells in the G1 phase, even with the rP21 pretreatment. However, rP21 also induces a slight increase in the G1 phase in MCF-10A when compared with CXCL12 (Figures 3A,C).

In the MDA-MB-231 treatment with rP21 or pretreatment with rP21 + CXCL12, the cells in the G1 phase increased by 51.52 and 53%, respectively, when compared to both the control and CXCL12 (Figures 3B,D). In the rP21 treatment, MDA-MB-231 cells also had an increase in the G1 phase when compared to CXCL12, which was significantly higher than in the MCF-10A cells (Figure 3E). In the rP21 pretreatment followed by CXCL12, MDA-MB-231 cells had an increase in the G1 phase when compared to CXCL12, which was also more significant than that in MCF-10A (Figure 3F). Thus, rP21 interferes in the cell cycle, increasing the G1 phase in MDA-MB-231, besides interfering with the CXCL12 action in these cells.

### rP21-Induced CXCR4 Internalization in MDA-MB-231

**FIGURE 4:**
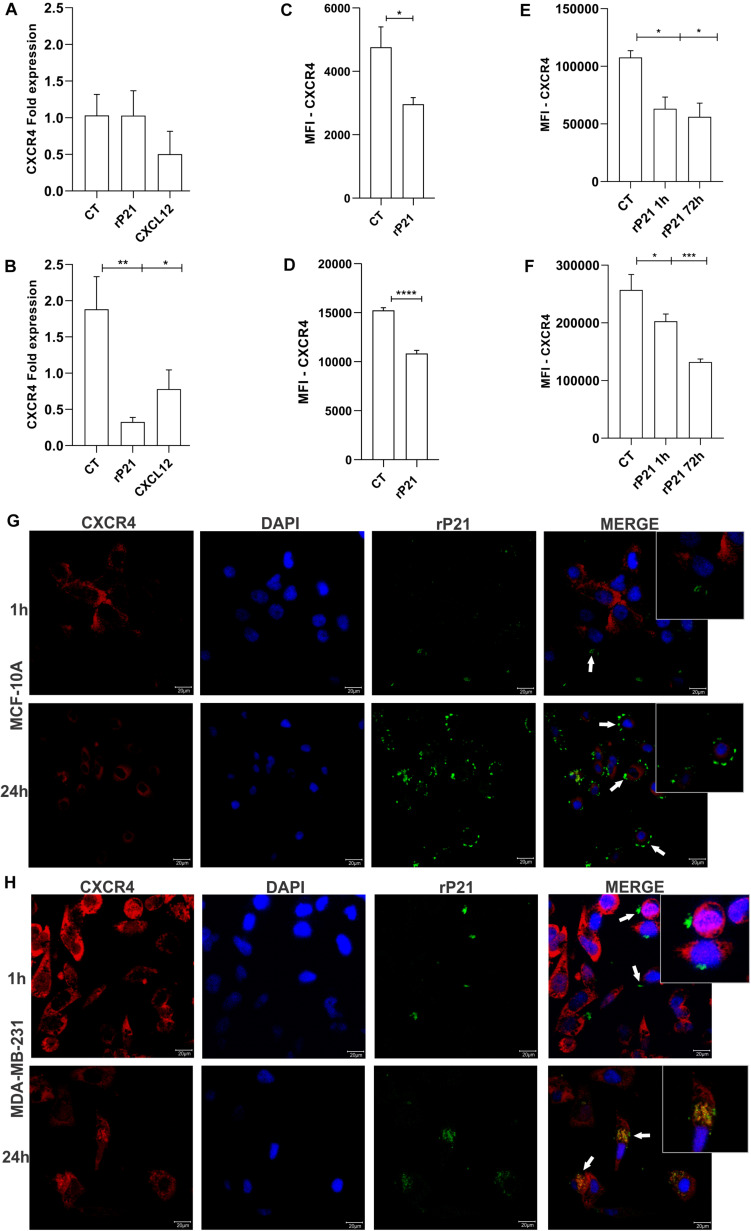
The rP21 protein downregulated CXCR4 gene expression, decreased the CXCR4 levels, and is internalized in MDA-MB-231. Gene expression of CXCR4 in MCF-10A **(A)** and MDA-MB-231 **(B)** after 72 h of treatment. Evaluation of CXCR4 in a membrane cell after rP21 treatment for 1 h in MCF-10A **(C)** and MDA-MB-231 **(D)** cells. Evaluation of CXCR4 in the cytoplasm after rP21 treatment for 1 and 72 h in MCF-10A **(E)** and MDA-MB-231 **(F)** cells. MCF-10A (**G**) and MDA-MB-231 **(H)** cells were treated with rP21 (100 μg/ml) for 1 and 24 h, fixed, permeabilized, rP21 (green) and CXCR4 (red) stained, and analyzed by confocal microscopy. Blue, cell nuclei. White arrows indicate the location of rP21. Bars, 20 μm represents × 63 objective. These results are representative of at least three independent experiments. Data show the mean ± SEM. Significant differences were determined using t-test. Differences were considered significant when *p* < 0.05. *p < 0.05, **p < 0.01, ***p < 0.001, and ****p < 0.0001.

To further gain insights into the MDA-MB-231-specific impact of rP21 in impairing cellular invasion and cell cycle, we analyzed CXCR4 after treatment with rP21. First, the CXCR4 gene expression was analyzed. In MCF-10A cells, rP21 and CXCL12 treatments did not alter CXCR4 expression. In MDA-MB-231, CXCL12 treatment downregulated the CXCR4 gene, but rP21 treatment downregulated the CXCR4 gene expression even less than CXCL12 (Figures 4A,B).

rP21 treatment for 1 h decreased the CXCR4 levels on the cell surface of MCF-10A (Figure 4C), in MDA-MB-231 this decreased is higher than in MCF-10A (Figure 4D), suggesting that rP21 binds in the same site as the anti-CXCR4 antibody or receptor internalization. The CXCR4 levels in the cytoplasm are also decreased. In MCF-10A, at both times, the decrease remains the same, being small relative to the control (Figure 4E). In MDA-MB-231, the decrease in CXCR4 increases over time (Figure 4F). A decreased CXCR4 labeling in the cytoplasm can indicate that rP21 may bind to the same region used by the antibody, or rP21 ligation in CXCR4 causes the internalization and desensitization of the receptors.

To explain these phenomena, rP21 binding kinetics were performed. The recombinant protein was adhered to the MCF-10A plasmatic membrane and not co-localized with CXCR4 at all times (Figure 4G). In contrast, in MDA-MB-231, rP21 co-localized with the CXCR4 receptor in 24 h, leaving the cell membrane surface and appearing in the cytoplasm, suggesting the internalization of P21 by its binding to the receptor (Figure 4H). In this way, rP21 binds to the CXCR4 receptor, and after 24 and 72 h of treatment, CXCR4 is internalized and its expression in the cytoplasm is lower in MDA-MB-231 than in MCF-10A cells. Besides that, rP21 downregulates CXCR4 gene expression in tumoral cells, but not in MCF-10A cells. Thus, the internalization of rP21 binding in the CXCR4 receptor interferes in tumor cell activity.

## Discussion

*Trypanosoma cruzi*-derived products have antitumor activities, such as a recombinant protein from GP82 (J18) that induces apoptosis in melanoma cells *in vitro* and reduces tumor *in vivo* (Atayde et al., 2008). Another product, calreticulin, is a calcium-binding protein from *T. cruzi* that can inhibit the activation of the complement cascade system favoring infection, besides the anti-angiogenic activity and antitumor properties *in vivo* (Valck et al., 2010; Ramírez et al., 2012).

P21 is an important protein during Chagas disease development (Rodrigues et al., 2012; Santos et al., 2014; Teixeira et al., 2015). The recombinant protein, rP21, has biological activities such as anti-angiogenic features (Teixeira et al., 2017) and promotes the chemotaxis of inflammatory cells (Teixeira et al., 2015) and actin cytoskeletal polymerization (Rodrigues et al., 2012). The rP21 protein did not interfere in cellular viability in any cell type, such as macrophages (Rodrigues et al., 2012), endothelial cell lines (Teixeira et al., 2017), myoblasts (Martins et al., 2020), and breast cells, such as the non-tumoral and tumoral cells shown here. Our results showed that rP21 binds to the CXCR4 receptors in MCF-10A and MDA-MB-231 cells and interferes in the migration/invasion and proliferation phenotypes of these cells. Corroborating these data, rP21 also binds to the CXCR4 of endothelial cells and inhibits vessel formation by inhibiting cell proliferation and increasing the cell numbers in the S phase (Teixeira et al., 2017).

CXCR4 is expressed in various cell types, including tumor cells. This receptor in prostate tumor cells, glioma, oral squamous carcinoma, pancreatic, and breast tumor cells is overexpressed when compared to non-tumor cells (Balkwill, 2004). Thus, CXCR4 can be studied as a potential therapeutic target for various types of tumors (Zhou et al., 2018). When the cells were treated with rP21, CXCR4 was downregulated. This downregulation of CXCR4 expression could also inhibit the distant metastasis of cancer (Sun et al., 2009). In MDA-MB-231, besides rP21, CXCL12 induces the downregulation of CXCR4. The constant presence of CXCL12 in the culture medium can induce the downregulation of CXCR4 and decrease the migration of these cells (Chatterjje and Gawaz, 2013).

In addition, to regulate CXCR4 gene expression, rP21 decreased the CXCR4 levels in the plasma membrane and cytoplasm of MDA-MB-231 cells, suggesting receptor internalization and desensitization. Our previous studies have shown that rP21 can bind to CXCR4 in HeLa cells, macrophages, and endothelial cells (Silva et al., 2009; Rodrigues et al., 2012; Teixeira et al., 2017). Pretreatment with rP21 in HeLa cells inhibited invasion by extracellular amastigote, suggesting that pre-incubation with rP21 could desensitize these cells (Silva et al., 2009). Thus, recombinant proteins can cause receptor desensitization to favor an infection, but they can cause receptor desensitization and generate beneficial effects in the tumor microenvironment. Chemokine internalization receptors occur after binders bind to a receptor. Depending on the receptor percentage being activated, this process may dramatically reduce the CXCR4 membrane expression levels and therefore change functionality (Neel et al., 2005). Internalization can cause receptor desensitization and unresponsiveness. CXCR4 desensitization in tumor cells is a potential therapy in TNBC and other overexpressed CXCR4 tumor cells. The CXCR4 antagonist AMD3100 is used in leukemia and solid tumor treatments to sensitize the cells to chemotherapy (Liu et al., 2016).

CXCR4 is implicated in promoting migratory phenotypes and proliferation in various types of tumors, and CXCL12 induces chemotactic response and cell proliferation (Tanaka et al., 2005). CXCR4-positive cells are driven by the CXCL12 concentration gradient; thus, tumoral cells leave the primary site toward organs that express more CXCL12, causing metastasis (Teicher and Fricker, 2010). rP21 treatment can modulate these phenomena and decrease invasion and proliferation, mainly in MDA-MB-231 cells. Besides, CXCL12-induced chemotaxis was reduced when cells were pretreated with rP21, showing that the recombinant protein blocks the CXCL12 effects. On the other hand, other drugs with cyclophosphamide, indicated for the treatment of cancerous diseases such as breast cancer and cervical cancer, induce the cell surface expression of CXCR4 and enhance the migration of MDA-MB-231 cells (Hung et al., 2017). Thus, CXCR4-positive cell invasion is dependent on CXCL12–CXCR4 binding. rP21 may bind to CXCR4 in a way that partially blocks CXCL12 downstream effects. The rP21 protein causes neutrophil and macrophage chemotaxis *in vivo* and *in vitro* (Teixeira et al., 2015) and has the same effect in MCF-10A cells, but our data showed that rP21 may act differently on tumor cells. This action in tumor cells can be explained by the rP21 downregulation of MMP-9 gene expression, while in MCF-10, the MMP-9 expression was not altered. MMP-9, as an indicator of migration, has been reported to be a downstream target of CXCL12/CXCR4 (Zuo et al., 2017). Thus, curcumin and luteolin impair the migratory activity in tumor cells by the repression of the CXCL12/CXCR4 biological axis, as a consequence of the downregulation of MMP-9 by luteolin (Qin et al., 2018; Bu and Zhao, 2020). Our results imply that rP21 repressed CXCL12-induced migration and invasion and decreased MMP-9 expression. However, in endothelial cells, rP21 upregulated the expression of MMP-9 after 72 h (Teixeira et al., 2017), but the role of MMP-9 during angiogenesis remains uncertain (Bendeck, 2004).

CXCR4 overexpression promotes migration, adhesion, and invasion in tumor cells, besides promoting epithelium–mesenchyme transition and acting in tumor development (Liu et al., 2016). Some compounds are capable of transforming normal cells, such as MCF-10A, altering important cell cycle proteins as well as inducing cell migration and invasion (Pierozan et al., 2018). Thus, rP21 does not interfere in the MCF-10A cell cycle, while this recombinant protein can arrest the TNBC cell cycle in the G1 phase. Cell cycle block is important in tumor cells because it may influence cell proliferation and migration (Kümper et al., 2016).

In this study, we demonstrate that the rP21 protein based on native *T. cruzi* reduces invasion, migration, and proliferation in MDA-MB-231 cells. The rP21 protein binds to the CXCR4 receptor in MCF-10A and MDA-MB-231, but this recombinant protein decreased the CXCR4 levels and was internalized only in tumoral cells. This internalization suggests that rP21 could desensitize CXCR4, triggering signaling events that decrease migration and invasion and, in addition, cause cell cycle arrest in tumoral breast cancer cells. The ability of rP21 to modulate cell cycle and invasiveness of breast malignant cell lines shed light on the potential use of this protein in triple-negative breast cancer therapeutic approaches to avoid tumor progression and metastasis.

## Data Availability Statement

The datasets generated for this study can be found in online repositories. The names of the repository/repositories and accession number(s) can be found below: http://dx.doi.org/10.14393/ufu.te.2019.1265.

## Author Contributions

BB was responsible for project development, designed the experimental approaches, performed the experimental manipulations, interpreted the data, and drafted the manuscript. IU, MAS, FM, FS, FL, AN, DL, ST, TT, and PC performed the experiments and participated in the data interpretation. IU designed the graphical abstract. AJ produced the rP21 anti-IgY and participated in the data interpretation. MC participated in the data interpretation and edited the manuscript. CS and MS coordinated and designed the biological experiments, analyzed and interpreted the data, and edited the manuscript. All authors read and approved the final manuscript.

## Conflict of Interest

The authors declare that the research was conducted in the absence of any commercial or financial relationships that could be construed as a potential conflict of interest.
