# Supplementary figures and images for: The Recombinant Protein Based on Trypanosoma cruzi P21 Interacts With CXCR4 Receptor and Abrogates the Invasive Phenotype of Human Breast Cancer Cells
Source: Front Cell Dev Biol. 2020 Oct 19;8:569729. doi: 10.3389/fcell.2020.569729 (PMC7604327; doi:10.3389/fcell.2020.569729)

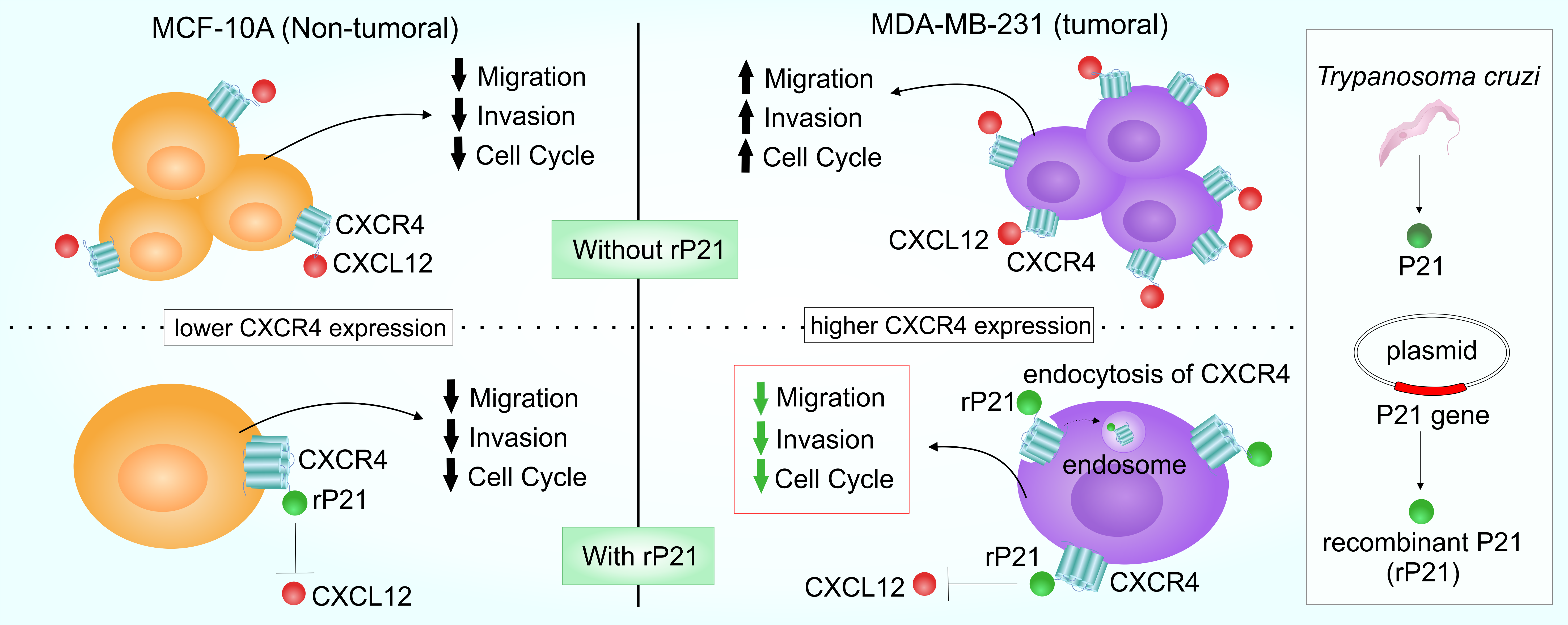

Supplement: Supplementary file 1 [file Image_1.TIF]

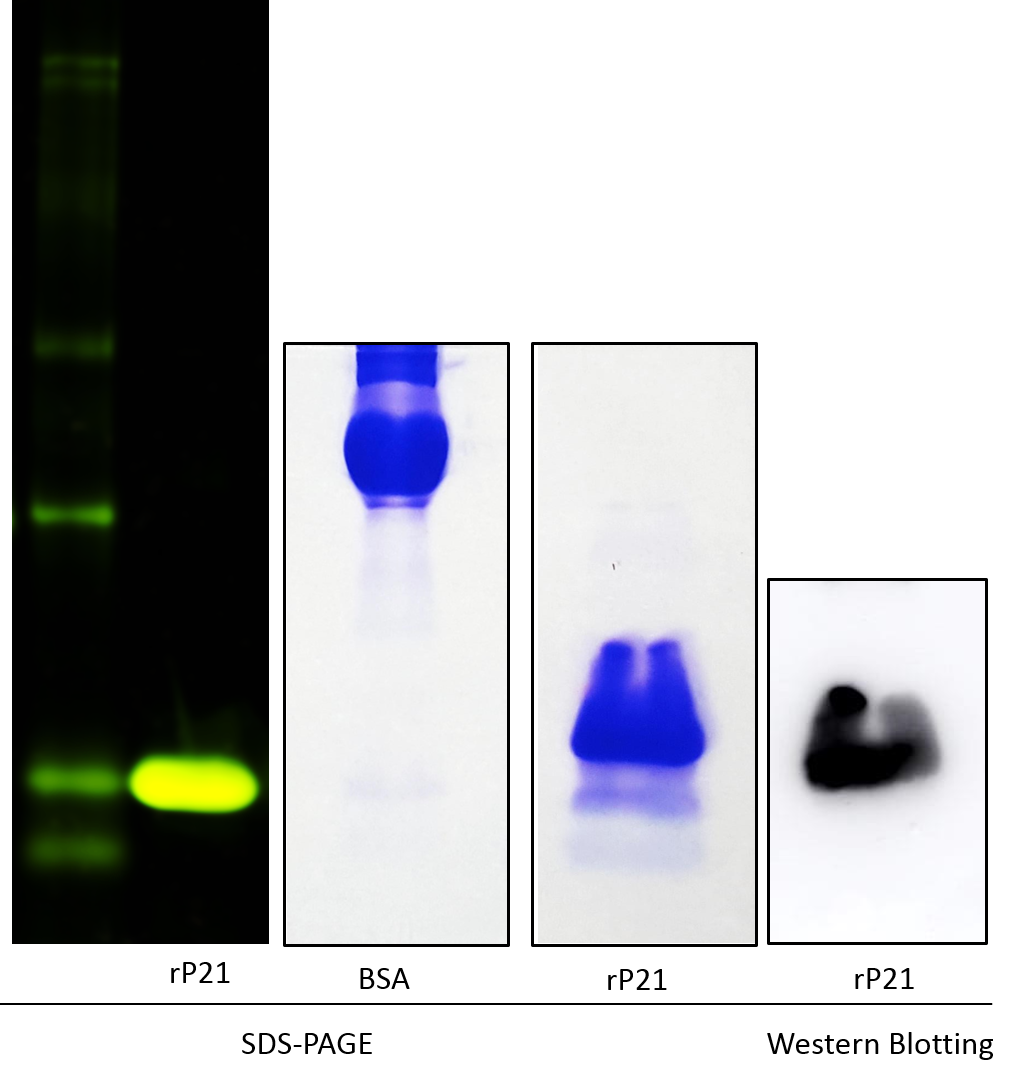

Supplement: Supplementary file 2 [file Image_2.TIF]
